# Genome-wide association analysis reveals *KCTD12* and miR-383-binding genes in the background of rumination

**DOI:** 10.1038/s41398-019-0454-1

**Published:** 2019-03-18

**Authors:** Nora Eszlari, Andras Millinghoffer, Peter Petschner, Xenia Gonda, Daniel Baksa, Attila J. Pulay, János M. Réthelyi, Gerome Breen, John Francis William Deakin, Peter Antal, Gyorgy Bagdy, Gabriella Juhasz

**Affiliations:** 1 Department of Pharmacodynamics, Faculty of Pharmacy, Semmelweis University, Budapest, Hungary; 2 NAP-2-SE New Antidepressant Target Research Group, Hungarian Brain Research Program, Semmelweis University, Budapest, Hungary; 3 Department of Measurement and Information Systems, Budapest University of Technology and Economics, Budapest, Hungary; 4 MTA-SE Neuropsychopharmacology and Neurochemistry Research Group, Hungarian Academy of Sciences, Semmelweis University, Budapest, Hungary; 5 Department of Psychiatry and Psychotherapy, Semmelweis University, Budapest, Hungary; 6 SE-NAP 2 Genetic Brain Imaging Migraine Research Group, Hungarian Brain Research Program, Semmelweis University, Budapest, Hungary; 7 NAP2 Molecular Psychiatry Research Group, Hungarian Brain Research Program, Semmelweis University, Budapest, Hungary; 8 Social, Genetic and Developmental Psychiatry Centre, King’s College London, London, UK; 9 Division of Neuroscience and Experimental Psychology, Faculty of Biology, Medicine and Health, University of Manchester, Manchester, UK; 10 Manchester Academic Health Sciences Centre, Manchester, UK; 11 Greater Manchester Mental Health NHS Foundation Trust, Prestwich, Manchester, M25 3BL UK

## Abstract

Ruminative response style is a passive and repetitive way of responding to stress, associated with several disorders. Although twin and candidate gene studies have proven the genetic underpinnings of rumination, no genome-wide association study (GWAS) has been conducted yet. We performed a GWAS on ruminative response style and its two subtypes, brooding and reflection, among 1758 European adults recruited in the general population of Budapest, Hungary, and Manchester, United Kingdom. We evaluated single-nucleotide polymorphism (SNP)-based, gene-based and gene set-based tests, together with inferences on genes regulated by our most significant SNPs. While no genome-wide significant hit emerged at the SNP level, the association of rumination survived correction for multiple testing with *KCTD12* at the gene level, and with the set of genes binding miR-383 at the gene set level. SNP-level results were concordant between the Budapest and Manchester subsamples for all three rumination phenotypes. SNP-level results and their links to brain expression levels based on external databases supported the role of *KCTD12*, *SRGAP3*, and *SETD5* in rumination, *CDH12* in brooding, and *DPYSL5*, *MAPRE3*, *KCNK3*, *ATXN7L3B*, and *TPH2* in reflection, among others. The relatively low sample size is a limitation of our study. Results of the first GWAS on rumination identified genes previously implicated in psychiatric disorders underscoring the transdiagnostic nature of rumination, and pointed to the possible role of the dorsolateral prefrontal cortex, hippocampus, and cerebellum in this cognitive process.

## Introduction

Ruminative response style refers to a trait-like tendency to reflect in a passive and repetitive way on personal feelings and difficulties^1,2^, being thus a manifestation of cognitive inflexibility and perseveration that prolongs the individual’s reaction to stress^3,4^. High scores on questionnaire measures of rumination are associated with increased risk of various mental disorders, including major depression, post-traumatic stress disorder, social phobia^1^, and with symptoms of alcohol abuse^5^, binge eating^6^, generalized anxiety^1^, and migraine^7^. By prolonging stress reaction it is thought to adversely affect cardiovascular and immune responses as well as numerous somatic complaints, such as pain^3,4^.

On the basis of factor analytic studies of questionnaire scales, Treynor et al.^8^ identified two subtypes of rumination: brooding, which denotes a maladaptive mechanism of passively comparing one’s current situation with an unachieved standard; and reflection which indicates a more adaptive strategy of purposefully turning inward for cognitive problem solving. According to twin studies among adolescents, rumination score has a 24% heritability^9^, ranging from 21% in case of the brooding to 37% in case of the reflection subtype^10^. However, in twin studies among young adults heritability of rumination is even higher, ranging from 34% for females to 40% for males^11^.

Candidate gene studies have revealed replicable associations with rumination. Three studies reported that the *5-HTTLPR* functional length polymorphism of the serotonin transporter gene *SLC6A4* promoter significantly interacts with life stress to increase rumination scores^12–14^. We demonstrated that the effect of the serotonin receptor 2A gene *HTR2A* on brooding is a function of childhood adversity^15^. Another study showed that the glucocorticoid receptor co-chaperone *FKBP5* gene interacts with attachment security to affect rumination scores in children^16^, and with childhood trauma to affect rumination in adolescents^17^. Our recent results have identified the *MTHFD1L* gene in the folate metabolism as a risk variant for rumination^18^. Furthermore, a gene–gene interaction effect on rumination has been reported for G protein-activated inwardly rectifying potassium channel subunit 2 (GIRK2) gene *KCNJ6* and cAMP-response element binding protein gene *CREB1*, pointing to the importance of synaptic plasticity in the generation of rumination^19^. Association of the brain-derived neurotrophic factor gene *BDNF* and rumination^20–22^ also points to this direction, although controversial results are available regarding rumination in adults^14,20,23^ and in children^22^. Despite its potential mediatory role in various disorders and the promising results of candidate gene studies, no genome-wide association studies (GWASs) have yet been reported for rumination.

In the present study, we performed a GWAS on rumination and its two subtypes, brooding and reflection, in a European general population to explore genetic risk variants and pathways that contribute to this cognitive phenotype.

## Methods

### Participants

This study was part of the NewMood study (New Molecules in Mood Disorders, Sixth Framework Program of the EU, LSHM-CT-2004-503474) and was funded by the European Union. All procedures were carried out in accordance with the Declaration of Helsinki, and were approved by the North Manchester Local Research Ethics Committee, Manchester, United Kingdom, and by the Scientific and Research Ethics Committee of the Medical Research Council, Budapest, Hungary. Participants aged between 18–60 years were recruited through advertisements, general practices, and a website in Greater Manchester, United Kingdom, and through advertisements and general practices in Budapest, Hungary. All of them provided a written informed consent, and all of them were of European white ethnic origin.

### Phenotype

Participants filled out the NewMood questionnaire pack, comprising the 10-item Ruminative Responses Scale (RRS)^8^, and a background questionnaire asking about gender, age, ethnicity, lifetime psychiatric problems, and present somatic disorders, relevant to rumination. RRS has two subscales, representing the two subtypes of rumination: brooding and reflection. We calculated the score for rumination, brooding and reflection as a continuous weighted score: the sum of item scores divided by the number of completed items.

### Genotyping, quality control, and imputation

Participants provided DNA by a genetic saliva sampling kit. Genomic DNA was extracted from buccal mucosa cells according to established protocols^24^. Genotyping was performed using Illumina's CoreExom PsychChip yielding a total of 573 141 variants, the genomic positions of which were defined according to the build GRCh37/hg19. Quality control and imputation was based on ref. ^25^, see also Supplementary File 1.

### Analyses

For descriptive statistics we used SPSS 25.

Our sample size greater than 200 enabled us to use parametric statistical methods, irrespectively of normality of distributions^26^.

To assess variance in each of the three examined traits explained by all single-nucleotide polymorphisms (SNPs) in our dataset we used the genomic-relationship-matrix restricted maximum likelihood (GREML) method in the genome-wide complex trait analysis (GCTA) software, version 1.26.0 (ref. ^27^). In the analysis of rumination, covariates were gender, age, and the first 10 calculated principal components (PCs) of the genetic data to correct for population substructure. In case of each subscale, the other subscale was also included as a further covariate, to eliminate their shared variance.

Primary SNP-based association tests for each phenotype were calculated using linear regression models in Plink 1.9 (https://www.cog-genomics.org/plink2), assuming an additive genetic effect. All models contained the covariates described above for the GCTA analyses.

To test the consistency and reproducibility of the above SNP-based association results between the Budapest and Manchester subsamples, sign tests were performed. First, SNPs were filtered in the combined Budapest + Manchester dataset by their *p*-values using a given threshold (*p* < 0.05, *p* < 1 × 10^–3^, and *p* < 1 × 10^–5^, respectively), based on refs. ^28,29^, and by their linkage disequilibrium (*R*^2^ ≥ 0.5) with the most significant SNP retained. For these remaining relevant and approximately independent SNPs, sign of the linear regression coefficients (betas) was compared between the Budapest and Manchester datasets. Rate of concordance with a 95% confidence interval and the *p*-value of the corresponding right-tailed sign test were calculated.

To carry all SNP-based associations to further levels, enrichment analysis was conducted both for individual genes and for gene sets. Gene-based annotations for SNPs were defined according to the RefGene database, build hg19, with an extension of 10,000 base pairs in both ends^30^. Gene sets were defined according to version 6.1 of MSigDB (http://software.broadinstitute.org/gsea/msigdb/), we examined sets defined in collections C5 (Gene Ontology—GO—terms, all categories), C3.mir (microRNA targets), and C3.tft (transcription factor targets). Gene sets were restricted to those containing at least 15 genes and no more than 300.

To aggregate *p*-values at the level of SNPs to the level of genes, the following methods were applied: (i) uncorrected minimal *p*-value within; (ii) minimal *p*-value adjusted according to Sidak's method; (iii) Fisher's method of combining correlated *p*-values modified according to Makambi^31^, and Kost and McDermott^32^; (iv) fixed-effect *z*-score statistics (http://www.biorxiv.org/content/biorxiv/early/2015/07/31/023648.full.pdf); also (v) a slightly modified version of Makambi's method (as implemented by the ‘--set-screen’ option of Plink); and (vi) the method described in ref. ^33^. These methods were also applied to sets of genes analogously treating the corresponding SNPs.

Furthermore, the effective chi-squared (ECS) and Gates^34^ methods (implemented by the software KGG 4.0^35^; version released on 8 September 2018) were also applied at the gene level, and, based on gene-level results of Gates, Wilcoxon and hybrid set-based test (HYST) methods (also implemented by KGG 4.0) were applied to derive results at the gene set level.

To further aggregate the above eight *p*-values (both at the gene and at the gene set level), the rank-averaging method described by the Psychiatric Genomics Consortium^36^ was applied in the permutation testing framework to yield a single (“empirical”) *p*-value and false discovery rate (FDR) *q*-value for each gene and gene set (with one million permutations). As in ref. ^36^, we consider the genes and gene sets with a *q* < 0.1 as significant.

To explore the known functional effects of our most significant SNPs as reported by public open databases, based on expression quantitative trait loci (eQTL) and 3D chromatin interaction, we used FUMA v1.3.1 (ref. ^37^), with a *p* ≤ 1x10^–5^ threshold for lead SNPs, an *R*^2^ ≥ 0.5 to define a genomic risk locus around a lead SNP, and a *p* ≤ 0.05 to involve SNPs into it. Each SNP of the genomic risk loci (referred to as top SNPs or our most significant SNPs) were mapped to a gene if either residing within gene boundaries extended by 10,000 base pairs, or having an FDR *q* ≤ 0.05 with it in the external eQTL, or a *q* ≤ 1 × 10^–6^ with its promoter region in the external chromatin interaction dataset^37^.

## Results

### Sample characteristics

After imputation and quality control steps, we had 3,474,641 SNPs and 1758 subjects (773 from Budapest and 985 from Manchester) with data on rumination, gender, age, and ten PCs of the genome. The number of SNPs entailed a Bonferroni-corrected significance threshold of *p* ≤ 1.44 × 10^–8^, and, at the SNP level, we considered *p* ≤ 1 × 10^–5^ a threshold for suggestive significance. Entering all SNPs into gene-based and gene set-based tests, our analyses yielded 25,371 genes, 4323 C5 gene sets, 182 C3 microRNA target (MIR) gene sets, and 550 C3 transcription factor target (TFT) gene sets.

Regarding descriptive statistics on rumination, gender, age, lifetime psychiatric problems, and present somatic disorders, Supplementary Table 1 shows that except for frequency of pain problems there are differences between the Budapest and Manchester subsamples in all variables at either a nominally significant (*p* ≤ 0.05) or trend (0.05 < *p* ≤ 0.10) level. The brooding and reflection subscales had a Pearson correlation of *r* = 0.488 (*p* < 0.00001) with each other in the combined sample, *r* = 0.373 (*p* < 0.00001) in Budapest, and *r* = 0.507 (*p* < 0.00001) in Manchester, underpinning the necessity of including the other subscale as a covariate when analyzing the specific variability of a subscale.

### SNPs in the background of rumination, brooding, and reflection

Before testing the role of particular SNPs in rumination, brooding, and reflection, we applied the GREML method to investigate the polygenic nature of these phenotypes, namely proportion of their variance residing in the whole set of the investigated SNPs, with results displayed in Table 1.

**Table 1:** Results of the GREML analysis for each phenotype and estimated SNP heritability

	Rumination	Brooding	Reflection
**Total variance**			
Value	0.294	0.325	0.324
Standard error of value	0.0100	0.0110	0.0110
**Variance explained by SNPs**			
Value	0.031	0.035	0.032
Standard error of value	0.0406	0.0397	0.0430
**SNP heritability**			
Value	0.105	0.107	0.099
Standard error of value	0.1380	0.1219	0.1328
*P*-value	0.230	0.164	0.226

*GREML* genomic-relationship-matrix restricted maximum likelihood method, *SNP* single-nucleotide polymorphism, *p*-value *p*-value of the respective model

**Fig. 1:**
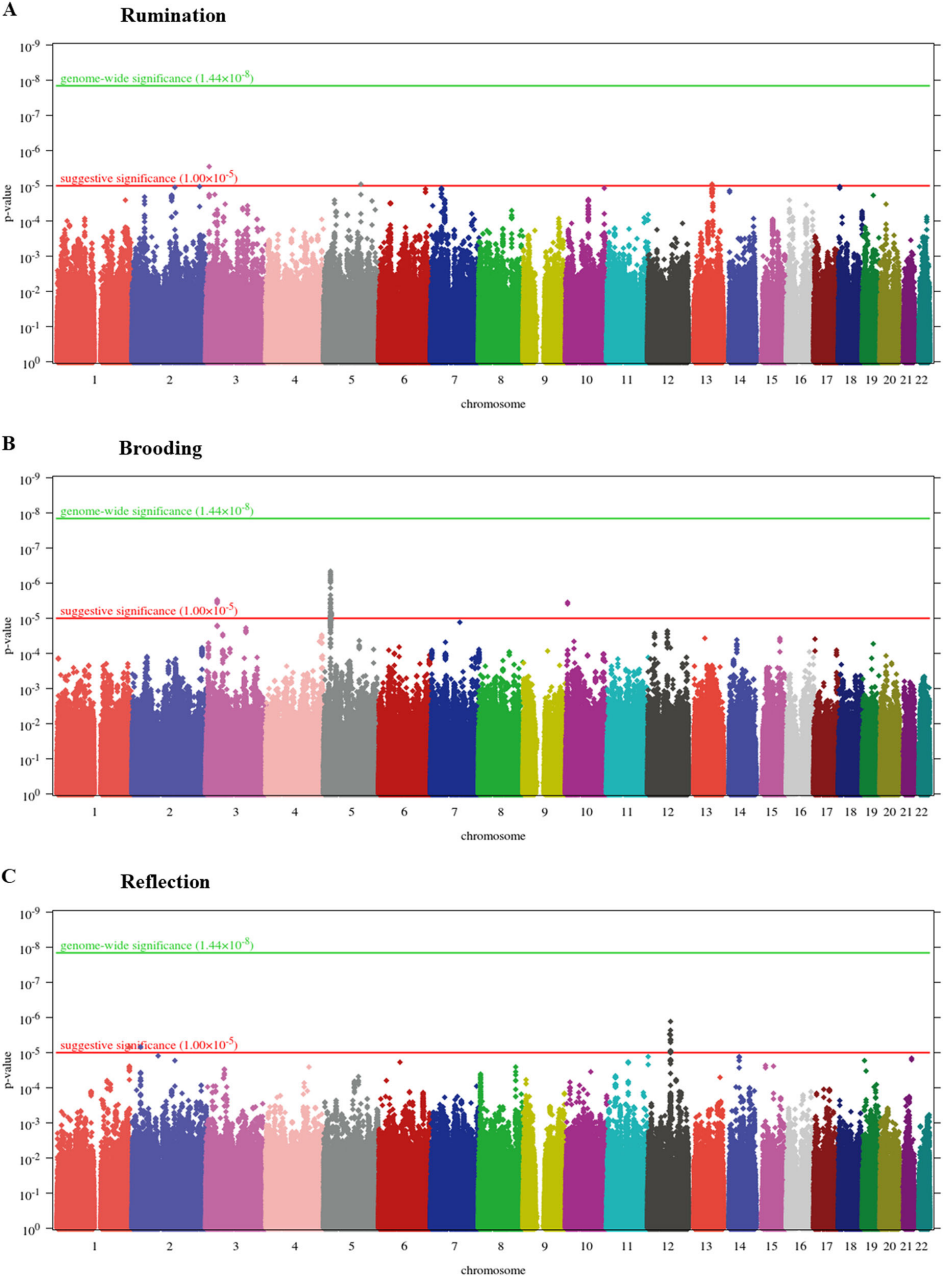
Manhattan plots of genome-wide SNP-based tests for rumination (**a**), brooding (**b**), and reflection (**c**) as outcome. *P*-value is displayed in function of genomic position for each single-nucleotide polymorphism (SNP). The red and green lines denote the levels of a suggestive and a genome-wide significance, respectively

With respect to particular SNPs, for rumination SNP-based association tests yielded a genomic inflation factor of λ = 1.00984. For the quantile-quantile (QQ) plot, see Supplementary Figure 1. No SNP survived Bonferroni correction for multiple testing but 3 SNPs had a suggestive significance which either reside in *LMCD1* or are intergenic (Fig. 1a and Supplementary Table 2).

**Fig. 2:**
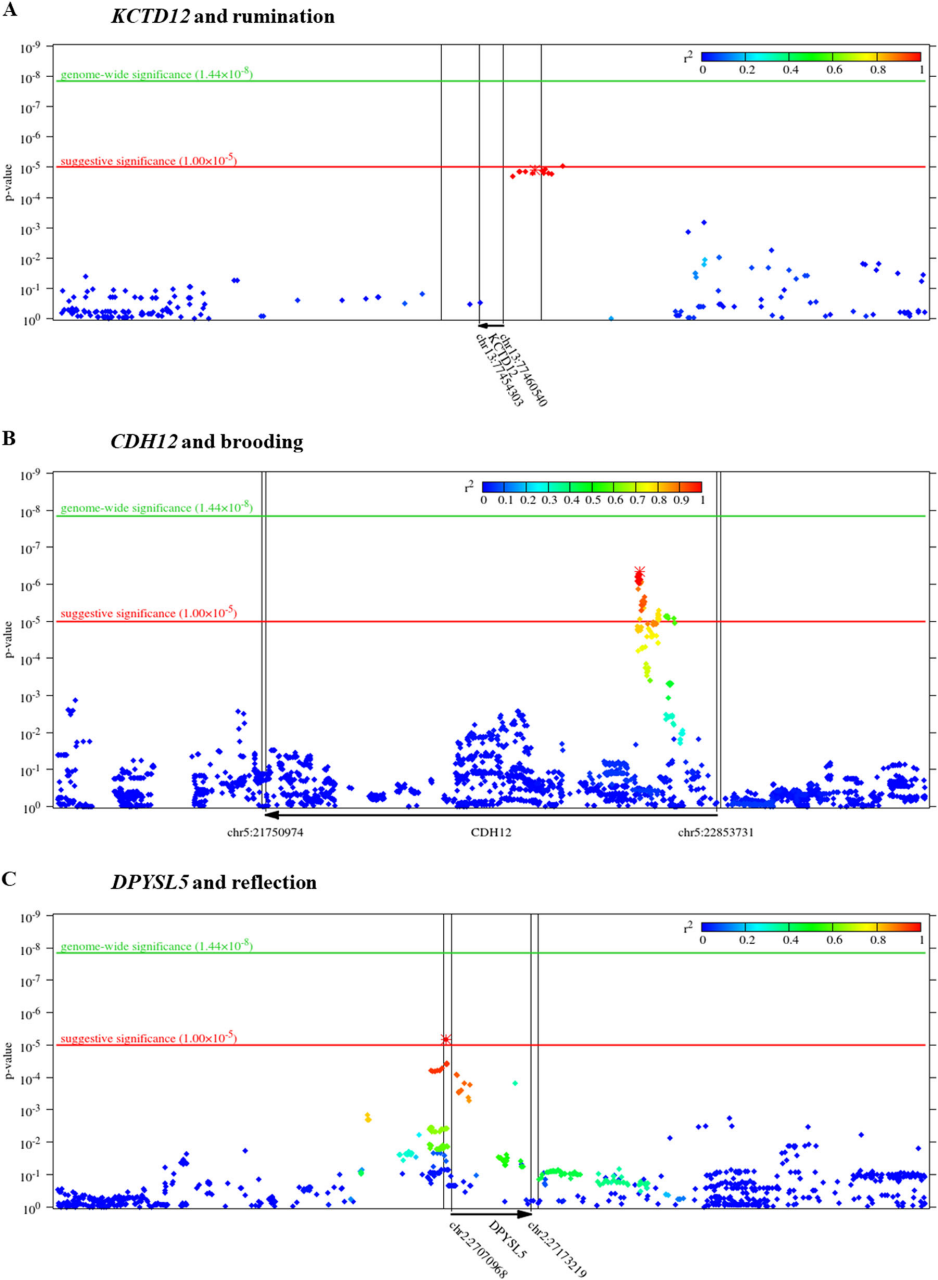
Zoomed Manhattan plots of *KCTD12* gene for rumination (**a**), *CDH12* gene for brooding (**b**), and *DPYSL5* gene for reflection (**c**) as outcome. *P*-value is displayed in function of genomic position for each single-nucleotide polymorphism (SNP) in the region. Colors denote the *r*^2^ value of linkage disequilibrium (LD) with the most significant SNP (marked with asterisk). Gene boundaries and their extension by 10,000 base pairs (as defined for the gene-based tests) are marked with vertical lines

In case of brooding, lambda value of the genome-wide SNP-based tests (for the QQ plot, see Supplementary Figure 2) was 1.00124. No SNP survived correction for multiple testing; however, we had 59 SNPs with suggestive significance (Fig. 1b and Supplementary Table 3). These SNPs are mapped to the *CDH12* (Fig. 2b), *STAC*, and *RBM17* genes.

Regarding reflection, according to the SNP-based tests, *λ* = 1. For the QQ plot, see Supplementary Figure 3. No SNP survived correction for multiple testing but we had 28 suggestively significant SNPs (Fig. 1c and Supplementary Table 4). Most of these 28 SNPs are intergenic within the chromosomal region of 12q21.1 (Supplementary Figure 4) but some of them reside in *DPYSL5* (Fig. 2c) or *CHRM3*.

Results of the sign tests on the reproducibility of SNP-based results between the Budapest and Manchester subsamples are displayed in Table 2 for all three phenotypes. We can see that the direction of effect of the independent lead SNPs was significantly concordant between the separate subsamples, except in case of the most stringent *p*-value threshold, which yields an inclusion of only one or two SNPs with a 100% but insignificant concordance.

**Table 2:** Results of sign tests on concordance of the independent lead SNPs between the Budapest and Manchester subsamples

*P*-value threshold for SNP inclusion		Rumination	Brooding	Reflection
0.05	Percentage of concordant SNPs (95% CI)	91.866% (91.413–92.302%) of 14,777 SNPs	93.687% (93.283–94.073%) of 14,826 SNPs	93.945% (93.551–94.323%) of 14,931 SNPs
	*P*-value of the sign test	<0.00001	<0.00001	<0.00001
1 ×10^–3^	Percentage of concordant SNPs (95% CI)	96.783% (94.448–98.327%) of 373 SNPs	96.296% (93.750–98.013%) of 351 SNPs	97.778% (95.668–99.036%) of 360 SNPs
	*P*-value of the sign test	<0.00001	<0.00001	<0.00001
1 ×10^–5^	Percentage of concordant SNPs (95% CI)	100% (2.500−100%) of 1 SNP	100% (15.811–100%) of 2 SNPs	100% (15.811–100%) of 2 SNPs
	*P*-value of the sign test	0.500	0.250	0.250

*P*-value threshold for SNP inclusion: refers to *p*-value of the SNP in the SNP-based linear regression model run in the combined Budapest + Manchester sample. *SNP* single-nucleotide polymorphism, *CI* confidence interval for the percentage of concordant SNPs, p-value of the sign test: significance of that the percentage of concordant SNPs deviate from that expected by chance

### Genes and gene sets in the background of rumination, brooding, and reflection

The top ten hits at the levels of genes and the three different gene sets are shown in Table 3. The complete list of these results and the intercorrelations between the respective methods are shown in Supplementary File 2, Supplementary File 3, and Supplementary File 4 for rumination, brooding, and reflection, respectively. The results for rumination demonstrate that after correction for multiple testing, *KCTD12* gene and the set of genes binding miR-383 survived the FDR *q* < 0.10 threshold. Figure 2a illustrates that *KCTD12* SNPs captured in our analysis reside exclusively in the upstream regulatory region of the gene. However, no gene or gene set survived the FDR *q* < 0.10 threshold for either brooding or reflection.

**Table 3:** Top ten hits for rumination, brooding, and reflection in the four categories

	Rumination			Brooding			Reflection		
Gene or gene set	Top ten hits	*p*-value	*q*-value	Top ten hits	*p*-value	*q*-value	Top ten hits	*p*-value	*q*-value
Genes	***KCTD12***	**<0.0001**	**0.0251**	*LOC284009*	<0.0001	0.1269	*C1QTNF5*	<0.0001	0.2308
	*KLHL33*	<0.0001	0.2263	*MFAP5*	<0.0001	0.1269	*USP2*	<0.0001	0.2308
	*TNFSF15*	<0.0001	0.2606	*CACNA1D*	0.0001	0.3592	*MFRP*	<0.0001	0.2308
	*UMOD*	0.0001	0.2606	*C7orf72*	0.0001	0.3592	*TOB1*	<0.0001	0.2308
	*MIR520F*	0.0001	0.2606	*VPS33B*	0.0001	0.3592	*TOB1-AS1*	<0.0001	0.2308
	*MIR519E*	0.0001	0.2606	*CTDSPL2*	0.0001	0.3592	*CADM4*	0.0001	0.2717
	*MIR515-1*	0.0001	0.2606	*S100A3*	0.0001	0.3592	*RNF26*	0.0001	0.2790
	*MIR515-2*	0.0001	0.2606	*EIF3J-AS1*	0.0001	0.3592	*DPYSL5*	0.0001	0.2790
	*MIR498*	0.0001	0.2606	*LOC101928034*	0.0001	0.3592	*ERCC1*	0.0001	0.2884
	*MIR520E*	0.0001	0.2606	*LOC101926911*	0.0001	0.3592	*CD3EAP*	0.0001	0.2884
C5	GO_LONG_TERM_MEMORY	0.0002	0.8149	GO_NOTCH_RECEPTOR_PROCESSING	<0.0001	0.2023	GO_PROXIMAL_DISTAL_PATTERN_FORMATION	0.0003	0.9746
	GO_EPHRIN_RECEPTOR_ACTIVITY	0.0004	0.8149	GO_NOTCH_BINDING	0.0003	0.5573	GO_RETINOL_METABOLIC_PROCESS	0.0006	0.9746
	GO_REGULATION_OF_DENDRITE_MORPHOGENESIS	0.0011	0.9213	GO_REGULATION_OF_MEMBRANE_REPOLARIZATION	0.0014	0.9719	GO_PANCREAS_DEVELOPMENT	0.0017	0.9746
	GO_EPHRIN_RECEPTOR_SIGNALING_PATHWAY	0.0013	0.9213	GO_CELLULAR_RESPONSE_TO_HEAT	0.0016	0.9719	GO_NEGATIVE_REGULATION_OF_LEUKOCYTE_MIGRATION	0.0022	0.9746
	GO_TRANSITION_METAL_ION_HOMEOSTASIS	0.0013	0.9213	GO_VOLTAGE_GATED_CALCIUM_CHANNEL_COMPLEX	0.0020	0.9719	GO_REGULATION_OF_RESPIRATORY_GASEOUS_EXCHANGE	0.0026	0.9746
	GO_REGULATION_OF_SYNAPTIC_VESICLE_TRANSPORT	0.0018	0.9213	GO_CALCIUM_CHANNEL_COMPLEX	0.0027	0.9719	GO_BASAL_LAMINA	0.0039	0.9746
	GO_POSITIVE_CHEMOTAXIS	0.0019	0.9213	GO_ENDOPLASMIC_RETICULUM_CALCIUM_ION_HOMEOSTASIS	0.0028	0.9719	GO_CYTOSOLIC_TRANSPORT	0.0043	0.9746
	GO_COLLAGEN_TRIMER	0.0020	0.9213	GO_POSITIVE_REGULATION_OF_KIDNEY_DEVELOPMENT	0.0029	0.9719	GO_POSITIVE_REGULATION_OF_SKELETAL_MUSCLE_TISSUE_DEVELOPMENT	0.0043	0.9746
	GO_GLYCOSAMINOGLYCAN_BINDING	0.0021	0.9213	GO_POSITIVE_REGULATION_OF_INTERLEUKIN_4_PRODUCTION	0.0037	0.9719	GO_PRIMARY_ALCOHOL_METABOLIC_PROCESS	0.0045	0.9746
	GO_CIRCADIAN_RHYTHM	0.0022	0.9213	GO_T_TUBULE	0.0038	0.9719	GO_MICROFILAMENT_MOTOR_ACTIVITY	0.0047	0.9746
C3 MIR	**TCTGATC_MIR383**	**0.0002**	**0.0442**	TCCAGAG_MIR518C	0.0062	0.8342	GCACCTT_MIR18A_MIR18B	0.0070	0.8978
	AACATTC_MIR4093P	0.0024	0.2196	ACTACCT_MIR196A_MIR196B	0.0136	0.8342	GAGCCAG_MIR149	0.0102	0.8978
	ACCATTT_MIR522	0.0126	0.7223	AGGGCCA_MIR328	0.0184	0.8342	ATTCTTT_MIR186	0.0149	0.8978
	ATTCTTT_MIR186	0.0209	0.7223	TCTGGAC_MIR198	0.0279	0.8342	CATTTCA_MIR203	0.0236	0.8978
	GGCCAGT_MIR193A_MIR193B	0.0213	0.7223	GAGCCTG_MIR484	0.0280	0.8342	TCTGATC_MIR383	0.0258	0.8978
	CAGTCAC_MIR134	0.0238	0.7223	GGGACCA_MIR133A_MIR133B	0.0338	0.8342	CCAGGGG_MIR331	0.0375	0.8978
	TCTGGAC_MIR198	0.0310	0.7223	CAGTCAC_MIR134	0.0359	0.8342	TCCAGAG_MIR518C	0.0419	0.8978
	TCCAGAG_MIR518C	0.0378	0.7223	CACCAGC_MIR138	0.0430	0.8342	CTTTGCA_MIR527	0.0504	0.8978
	AGTCAGC_MIR345	0.0401	0.7223	TACTTGA_MIR26A_MIR26B	0.0437	0.8342	ATAACCT_MIR154	0.0507	0.8978
	AAACCAC_MIR140	0.0423	0.7223	ATGCTGC_MIR103_MIR107	0.0500	0.8342	GGTAACC_MIR4095P	0.0532	0.8978
C3 TFT	PAX4_03	0.0026	0.7520	SF1_Q6	0.0014	0.6614	ATCMNTCCGY_UNKNOWN	0.0008	0.4565
	MTF1_Q4	0.0032	0.7520	MZF1_01	0.0024	0.6614	ACAWYAAAG_UNKNOWN	0.0023	0.6212
	OCT1_B	0.0056	0.7520	CACBINDINGPROTEIN_Q6	0.0045	0.7805	FOXO4_01	0.0042	0.7784
	GGGNRMNNYCAT_UNKNOWN	0.0080	0.7520	FAC1_01	0.0073	0.7805	FXR_IR1_Q6	0.0081	0.9499
	NF1_Q6_01	0.0082	0.7520	TGANNYRGCA_TCF11MAFG_01	0.0084	0.7805	TGATTTRY_GFI1_01	0.0113	0.9499
	TCF11MAFG_01	0.0096	0.7520	NFY_C	0.0087	0.7805	E2F1_Q3	0.0117	0.9499
	IK3_01	0.0106	0.7520	OSF2_Q6	0.0133	0.7805	E2F_Q6	0.0121	0.9499
	IRF7_01	0.0109	0.7520	AP4_Q6	0.0142	0.7805	E2F_Q4	0.0155	0.9517
	E2F1_Q4	0.0175	0.9271	AP1_Q2_01	0.0148	0.7805	NFY_Q6_01	0.0162	0.9517
	RP58_01	0.0199	0.9271	TAAYNRNNTCC_UNKNOWN	0.0169	0.7805	TTGCWCAAY_CEBPB_02	0.0202	0.9517

C5: GO gene sets; C3 MIR and C3 TFT: the two motif gene set collections, grouping genes by microRNA target sites or transcriptional factor binding sites, respectively; *p*-value: empirical *p*-value based on one million permutations; *q*-value: false discovery rate correction, done for all analyses within each of the four categories, for each phenotype. Significant hits are marked with bold

### Functional effects of the top SNPs on gene expression regulation in the brain

Supplementary Figures 5–11 show FUMA^37^ results on the genes regulated in brain by the top SNPs according to external chromatin interaction databases^38^ and the following eQTL databases. GTEx v6 and v7 (refs. ^39,40^) and BRAINEAC^41^ comprise several brain regions. However, xQTLServer^42^ and CommonMind Consortium (CMC)^43^ samples encompass only the dorsolateral prefrontal cortex (DLPFC). FUMA results on the regulated genes in all available tissues and cell types without restriction to brain are displayed in Supplementary Files 2–4 for each phenotype.

Results revealed that top SNPs for rumination on chromosome 3 were associated with expression levels of *SRGAP3* and *SETD5* in the DLPFC (CMC samples) (Supplementary Figure 5 and Supplementary File 2). Top rumination SNPs on chromosome 13 influenced expression level of *KCTD12* also in the DLPFC (CMC). They also interacted with *C13orf45* (*LMO7DN*) in the hippocampus, DLPFC, and neural progenitor cells (Supplementary Figure 7 and Supplementary File 2).

Most significant SNPs for brooding affected expression level of *CDH12* in the DLPFC (CMC) (Supplementary Figure 8 and Supplementary File 3).

Top SNPs for reflection on chromosome 2 had many effects according to external databases (Supplementary Figure 10 and Supplementary File 4). In the DLPFC they altered expression levels of *DPYSL5* (CMC and xQTLServer), *SLC35F6*, *FNDC4*, *MAPRE3* (CMC), and *KCNK3* (xQTLServer). In the BA9 region they affected expression levels of *GPN1* (GTEx v6) and also *KCNK3* (GTEx v7). In the cortex in general, they regulated expression levels of *DPYSL5* (GTEx v6 and v7) and *KCNK3* (GTEx v7). Furthermore, they altered *DPYSL5* expression in hippocampus, substantia nigra (GTEx v7), cerebellum, cerebellar hemisphere (GTEx v6 and v7), and white matter (BRAINEAC).

Reflection top SNPs on chromosome 12 influenced expression level of *ATXN7L3B* in the inferior olivary nucleus (BRAINEAC), and also took part in chromatin interactions with *TPH2* and *TRHDE* in neural progenitor cells (Supplementary Figure 11 and Supplementary File 4).

## Discussion

We present a GWAS of ruminative response style and its two subtypes. The association of *KCTD12* gene and miR-383-binding genes with rumination appears to be robust because these results survived correction for multiple testing. We discuss the implications for the biological foundations of rumination below. While previously reported candidate gene results were not replicated at the more stringent genome-wide level, new candidate genes emerged in our study.

In spite of their diversity, we only discuss three aspects of our findings: (i) transdiagnostic nature of rumination, (ii) relevant brain regions in rumination, and (iii) poligenicity of rumination.

### Transdiagnostic nature of rumination, brooding, and reflection, supported by *KCTD12*, miR-383, and suggestively significant SNPs

*KCTD12*, significant at gene level in the present study, emerged as a candidate in a bipolar depression GWAS among Han Chinese^44^. Rumination has indeed been suggested to show higher levels in bipolar than in major depressive patients^45^, and to be independent of bipolar patients’ mood state^46^.

Suggestively significant SNPs for brooding regulated brain expression level of *CDH12*, which result corroborates the genetic relationship of rumination phenotypes with bipolar depression and extends it to other disorders. *CDH12* has been previously associated with bipolar depression, major depression, and schizophrenia^47,48^, and also with bipolar-type schizoaffective disorder^49^, suicidal behavior^50,51^, and metamphetamine and alcohol dependence^48^. *CHRM3* gene, highlighted by a suggestive SNP for reflection, has also been implicated in schizophrenia^52^ but binding results of its encoded protein, muscarinic acetylcholine receptor M3, are conflicting with regard to bipolar and major depressive patients^53,54^. A robust evidence underpins the role of rumination in major depression^55^, and there is evidence on its relevance also in psychosis^56^, alcohol abuse^5^, and substance abuse^6^.

Underscoring the genetics-based importance of rumination phenotypes in suicidality, the tryptophan hydroxylase *TPH2* gene, implicated in chromatin interactions of SNPs suggestive for reflection, is related to hopelessness, a suicidality risk phenotype^57^. It has to be noted, however, that rumination and brooding have shown a more consistent positive association with suicide phenotypes than reflection^58^.

The set of genes binding miR-383, significant for rumination after correction for multiple testing, can also be settled in this transdiagnostic context, specifically that of stress and binge eating which can be interpreted as a cause and a possible consequence of rumination, respectively^1,2,6^. MiR-383 expression has been revealed to be upregulated in the rat serum after chronic unpredictable mild stress^59^, and in the hypothalamus of mice deficient of either leptin or leptin receptor, with an intraperitoneal injection of leptin downregulating its expression^60^. Serum leptin levels have shown conflicting associations with binge eating symptoms^61,62^. Nevertheless, direction of effects with regard to miR-383 and rumination needs to be investigated by future studies.

The 12q21.1 region, comprising suggestively significant SNPs for reflection, has been associated with mental retardation^63^. Top SNPs for rumination also underpin the genetic link with mental retardation, since they affected brain expression levels of *SRGAP3* and *SETD5*, implicated in this disorder^64,65^, but see ref. ^66^. However, rumination has shown a positive correlation only with verbal but not with non-verbal intelligence scores^67^.

While these disorders represent diverse phenotypes, based on these overlapping genetic results we propose rumination as an overarching trait, sharing biological underpinnings with several psychiatric disorders.

### Relevant brain regions in rumination, brooding, and reflection, based on gene regulation databases and previous literature

Although the present results do not provide direct evidence that the implicated genes exert their effect on rumination and its subtypes via their expression in certain brain regions, we discuss three regions most salient from our results: DLPFC, hippocampus, and cerebellum. The role of the DLPFC^68^ and hippocampus^69,70^ has been suggested in rumination, but results on the role of the cerebellum have yielded contradictory associations^71,72^. Nevertheless, several other brain regions or even other tissues may play a role in mediating between these genes and rumination but they are not discussed here.

While we demonstrated that the expression of our significant gene, *KCTD12*, was regulated by our top SNPs only in DLPFC, its relevance has been suggested in the hippocampus and the cerebellum by previous literature. For example, Kctd12-KO mice showed an increased intrinsic excitability of pyramidal neurons in the hippocampus in addition to an increased fear-learning phenotype^73^. *KCTD12* encodes an auxiliary subunit exclusively associated with the GABA_B_ receptor^74^. The encoded protein enhances receptor signaling at the cell surface^75^ and rapidly desensitizes the K^+^ current response mediated by Kir3 channels after GABA_B_ activation^74,76^. The Kir3.2 (GIRK2) subunit of Kir3 channels is encoded by the *KCNJ6* gene associated with rumination in our previous results^19^. GABA_B_ and GIRK2 are co-localized^77^ and have a concerted action in the hippocampus^78–80^ and in cerebellar Purkinje cells^81^. Antagonism of the GABA_B_ receptor has been suggested to have antidepressant properties^82,83^, and rapid antidepressants may act through decoupling GABA_B_ from the Kir3 channel via the adaptor protein 14-3-3eta^80^, highlighting the importance of Kir3 activation among the numerous downstream effects of GABA_B_ in current depression level. On the other hand, the possible action of *KCTD12* on rumination can also be viewed from a developmental perspective, since it showed extremely low expression in the adult cerebrum and cerebellum but high brain expression levels in the fetal stages in a study^84^. This may resolve contradictions concerning brain regions between our present results and previous literature to some extent.

Nevertheless, top SNPs of both *KCNK3*, suggested in astrocytes of temporal lobe epilepsy patients’ hippocampus^85^, and *MAPRE3*, implicated in dendritic spine morphology and synaptic plasticity in mature hippocampal neurons^86^, have affected their expression levels only in the DLPFC or cortex in our results but not in the hippocampus.

With regard to the cerebellum, *RBM17* emphasizes Purkinje neurons^87^. The 12q21.1 region has been linked to cerebellar ataxia^88^, and specifically the *ATXN7L3B* (*lnc-SCA7*) gene within has been proposed to have a role in spinocerebellar ataxia^89^. However, our top SNPs influenced *ATXN7L3B* expression within the inferior olivary nucleus.

In contrast to the controversies detailed above, *DPYSL5* (or *CRMP5*), a gene implicated in reflection in our results, yielded consistent associations between expression databases and previous literature, stressing the importance of the hippocampus and the cerebellum. It is involved in brain development and in adult neurogenesis^90^, in addition to the dendrite morphology and synaptic plasticity of cerebellar Purkinje cells^91^. In mouse embryonic hippocampal neurons DPYSL5 inhibits neurite outgrowth^92^, dendrite outgrowth and formation^93^, and decreases mitochondrial content in dendrites^94^, again pointing to a possible critical window of rumination establishment during fetal development of the hippocampus.

To summarize, there are both consistencies and inconsistencies between gene regulation databases and previous literature regarding these three most salient brain regions in our results.

### Polygenicity of rumination, brooding, and reflection

No SNP association survived correction for multiple testing but there were several suggestively significant results.

Lack of significance both in SNP-based association tests and SNP heritability may be the consequence of the statistical power of our study, because of the weak effects, and limited sample size in relation to a large number of SNPs^95^. However, the lack of power is offset by our replication subsamples from Budapest and Manchester. The sign test analysis demonstrated the replicability of the effects of independent lead SNPs: the rate of concordant SNPs significantly deviated from that expected by chance both for the SNPs with *p*-values less than 0.05 and 1 × 10^–3^. However, this deviation was not significant for the most significant (*p* < 1 × 10^–5^) very few SNPs. This genetic concordance is also remarkable because the two subsamples differed from each other not only in rumination levels but also in frequencies of most disorders related to rumination.

## Limitations

Although testing at multiple levels and utilizing external databases of gene expression and chromatin interaction convey strengths to our study, one of its weaknesses is the low sample size^29^. This not only limits the power of our tests, but also explains that we chose mega-analysis instead of meta-analysis, despite differences in the rumination phenotypes between the two subsamples.

Another limitation is that we measured rumination with only one method, thus we were not able to create any latent rumination variable, like Johnson et al.^11^ did with RRS brooding, RRS reflection, and the rumination component of the Rumination-Reflection Questionnaire. Genome-wide investigation of other rumination measurements, as well as GWASes within specific subpopulations, such as depressed patients, would also be inevitable.

## Conclusions

Although our present study is limited by its low sample size, the replicability of the effects of independent lead SNPs between the two subsamples is remarkable given the phenotypic differences between them. This underlines the robustness of the genetic background of rumination across European populations.

The genetically underpinned overarching nature of the rumination endophenotype implies its clinical relevance in several fields.

Further studies are needed to shed light on the mediating pathways between the implicated genes and rumination. Developmental and adult perspectives can be highlighted in the association of rumination with specific brain regions, such as DLPFC, hippocampus, and cerebellum. A possible cooperation of KCTD12, GIRK2, and GABA_B_ receptor proteins should also be clarified in the future.

## Supplementary information

Supplementary File 1

Supplementary File 2

Supplementary File 3

Supplementary File 4

Supplemental legends
