# Supplementary material for: Genome-wide association analysis reveals KCTD12 and miR-383-binding genes in the background of rumination
Source: Transl Psychiatry. 2019 Mar 18;9:119. doi: 10.1038/s41398-019-0454-1 (PMC6423133; doi:10.1038/s41398-019-0454-1)
Supplement: Supplementary file 1 [file 41398_2019_454_MOESM1_ESM.docx]

**Methods**

**Quality control and imputation**

The set of variants were imputed and filtered according to multiple quality control (QC) steps. The steps applied were the following.

Prior to imputation, single-nucleotide biallelic variants of autosomal chromosomes were extracted from the dataset, and their strand alignments were checked against that of the reference data provided by IMPUTE2 (see <https://mathgen.stats.ox.ac.uk/impute/1000GP_Phase3.html>). Imputation was performed using a two-phase process: first haplotype information was determined using SHAPEIT (<https://mathgen.stats.ox.ac.uk/genetics_software/shapeit/shapeit.html>), then missing variants were imputed from the reference data using IMPUTE2 (<http://mathgen.stats.ox.ac.uk/impute/impute_v2.html>), yielding a total of 81,613,199 variants.

The subsequent QC consisted of the following steps. First, multiallelic and not single-nucleotide variants were excluded, and second, we excluded variants with an imputation score “info” less than 0.5 or “certainty” less than 0.7. After that, filtering of variants and participants was performed separately for each of the combined Budapest + Manchester sample, the Budapest subsample and the Manchester subsample. Thresholds for these QC steps were the following: a minor allele frequency (MAF) minimum of 0.01; iteratively 0.1, 0.05, and 0.01 missingness; a p-value ≥ 1x10^-5^ for the Hardy-Weinberg equilibrium test; an R^2^ of 0.2 for the linkage disequilibrium (LD) pruning; and an identical-by-descent π^^^ ≤ 0.1875. We further excluded individuals with a problematic inferred gender, or those who were outliers according to their heterozygosity.

**Results**

**Descriptive statistics**

|  |  | **Budapest+Manchester** | | **Budapest** | | **Manchester** | | **Difference between Budapest and Manchester** | |
| --- | --- | --- | --- | --- | --- | --- | --- | --- | --- |
|  |  | **Mean** | **S.E.M.** | **Mean** | **S.E.M.** | **Mean** | **S.E.M.** | **t** | **p** |
| **Rumination score** |  | 2.119 | 0.0137 | 1.943 | 0.0168 | 2.257 | 0.0195 | -12.214 | <0.00001 |
| **Brooding score** |  | 2.146 | 0.0161 | 1.934 | 0.0198 | 2.313 | 0.0228 | -12.554 | <0.00001 |
| **Reflection score** |  | 2.092 | 0.0156 | 1.952 | 0.0208 | 2.202 | 0.0221 | -8.229 | <0.00001 |
| **Age** |  | 32.572 | 0.2498 | 30.757 | 0.3730 | 33.997 | 0.3294 | -6.514 | <0.00001 |
|  |  | **Frequency** | **%** | **Frequency** | **%** | **Frequency** | **%** | **χ^2^** | **p** |
| **Gender** | Male | 503 | 28.612% | 240 | 31.048% | 263 | 26.701% | 4.008 | 0.045 |
|  | Female | 1255 | 71.388% | 533 | 68.952% | 722 | 73.299% |  |  |
| **Depression** | Not reported | 1039 | 59.101% | 607 | 78.525% | 432 | 43.858% | 215.343 | <0.00001 |
|  | Reported | 719 | 40.899% | 166 | 21.475% | 553 | 56.142% |  |  |
| **Suicide attempt or deliberate self-harm** | Not reported | 1550 | 88.168% | 736 | 95.213% | 814 | 82.640% | 65.641 | <0.00001 |
|  | Reported | 208 | 11.832% | 37 | 4.787% | 171 | 17.360% |  |  |
| **Manic episode, manic depression or bipolar disorder** | Not reported | 1710 | 97.270% | 762 | 98.577% | 948 | 96.244% | 8.879 | 0.003 |
|  | Reported | 48 | 2.730% | 11 | 1.423% | 37 | 3.756% |  |  |
| **Anxiety, panic or phobia** | Not reported | 1298 | 73.834% | 624 | 80.724% | 674 | 68.426% | 33.906 | <0.00001 |
|  | Reported | 460 | 26.166% | 149 | 19.276% | 311 | 31.574% |  |  |
| **Obsessive-compulsive disorder** | Not reported | 1705 | 96.985% | 758 | 98.060% | 947 | 96.142% | 5.446 | 0.020 |
|  | Reported | 53 | 3.015% | 15 | 1.940% | 38 | 3.858% |  |  |
| **Psychotic episode or schizophrenia** | Not reported | 1741 | 99.033% | 769 | 99.483% | 972 | 98.680% | 2.911 | 0.088 |
|  | Reported | 17 | 0.967 | 4 | 0.517% | 13 | 1.320% |  |  |
| **Eating disorder** | Not reported | 1602 | 91.126% | 720 | 93.144% | 882 | 89.543% | 6.943 | 0.008 |
|  | Reported | 156 | 8.874 | 53 | 6.856% | 103 | 10.457% |  |  |
| **Drug or alcohol problem** | Not reported | 1664 | 94.653% | 757 | 97.930% | 907 | 92.081% | 29.276 | <0.00001 |
|  | Reported | 94 | 5.347% | 16 | 2.070% | 78 | 7.919% |  |  |
| **Cardiovascular disorder** | Not reported | 1714 | 97.497% | 748 | 96.766% | 966 | 98.071% | 3.024 | 0.082 |
|  | Reported | 44 | 2.503% | 25 | 3.234% | 19 | 1.929% |  |  |
| **Pain problem (migraine, back pain, other pain, rheumatoid arthritis)** | Not reported | 1503 | 85.495% | 664 | 85.899% | 839 | 85.178% | 0.182 | 0.670 |
|  | Reported | 255 | 14.505% | 109 | 14.101% | 146 | 14.822% |  |  |

***Supplementary Table 1.* Descriptive statistics for our sample, recruited in the general population. Psychiatric problems denote a lifetime problem (“Have you ever had…”), and the two somatic ones denote a present problem (“Do you have…”).** S.E.M.: standard error of mean; t: test statistic of t test; χ^2^: Pearson chi-square; p: p-value.

**SNP-based results for rumination**

**
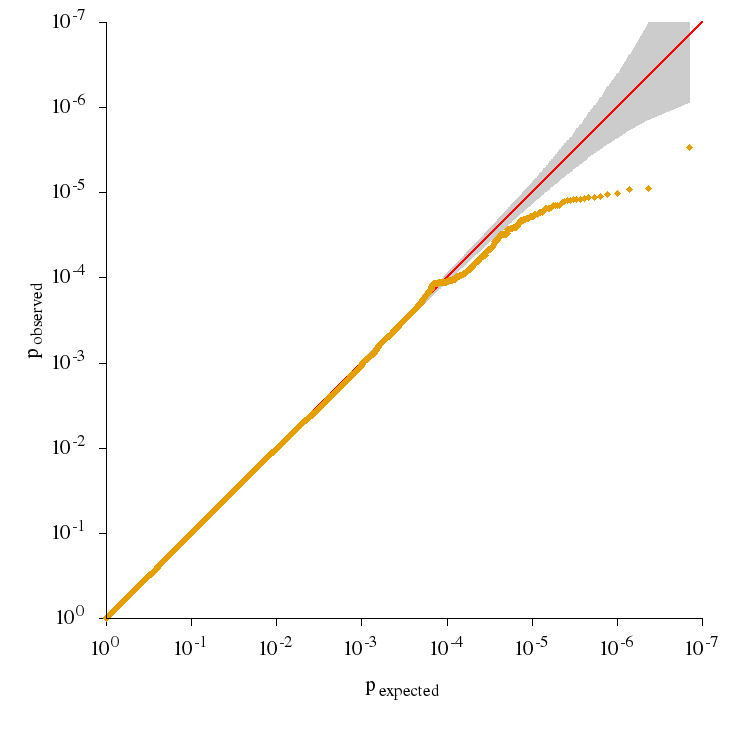
**

***Supplementary Figure 1*. Quantile-quantile plot of genome-wide SNP-based tests for rumination as outcome, with a 95% confidence interval marked.** SNP: single-nucleotide polymorphism.

| **Chr** | **Gene** | **SNP** | **Bp** | **A1** | **Beta** | **P** |
| --- | --- | --- | --- | --- | --- | --- |
| 3 | *LMCD1* | rs114122346 | 8578294 | T | -0.2038 | 2.91E-06 |
| 13 | intergenic | rs674041 | 77475971 | T | -0.0937 | 8.90E-06 |
| 5 | intergenic | rs150429966 | 120510351 | G | 0.307 | 9.05E-06 |

***Supplementary Table 2.* SNPs with a suggestive significance (p≤1x10^-5^) for rumination as outcome.** Chr: chromosome; SNP: single-nucleotide polymorphism; bp: base position; A1: minor allele; p: p-value. Genes based on UCSC Genome Browser, GRCh37/hg19: <https://genome.ucsc.edu/>

**SNP-based results for brooding**

**
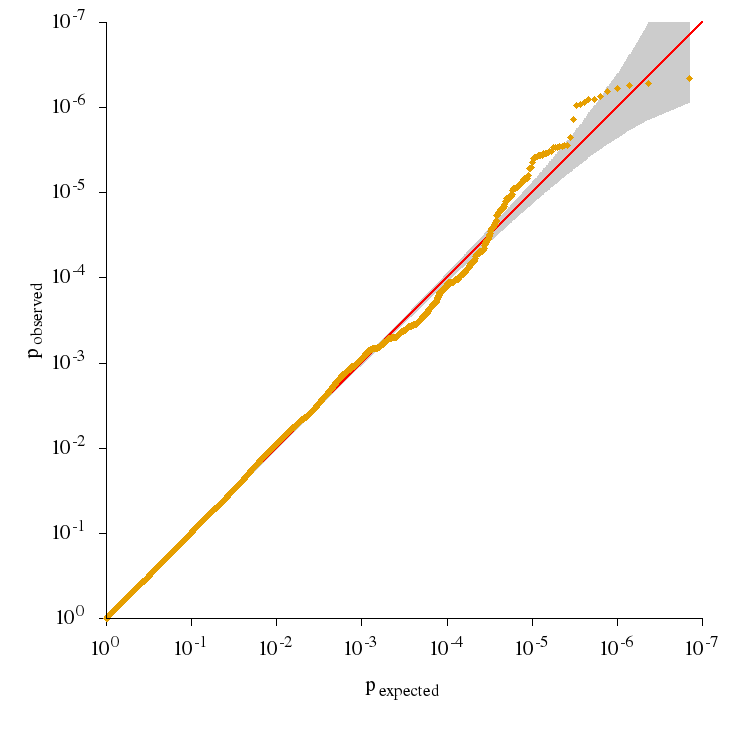
**

***Supplementary Figure 2*. Quantile-quantile plot of genome-wide SNP-based tests for brooding as outcome, with a 95% confidence interval marked.** SNP: single-nucleotide polymorphism.

| **Chr** | **Gene** | **SNP** | **Bp** | **A1** | **Beta** | **P** |
| --- | --- | --- | --- | --- | --- | --- |
| 5 | *CDH12* | rs1545967 | 22664795 | A | 0.1028 | 4.52E-07 |
| 5 | *CDH12* | rs6872869 | 22661088 | G | 0.1025 | 5.16E-07 |
| 5 | *CDH12* | rs10061490 | 22665159 | A | 0.1022 | 5.49E-07 |
| 5 | *CDH12* | rs6882492 | 22664430 | A | 0.1018 | 5.98E-07 |
| 5 | *CDH12* | rs6452087 | 22660431 | T | 0.1015 | 6.49E-07 |
| 5 | *CDH12* | rs1487410 | 22668104 | A | 0.1009 | 7.33E-07 |
| 5 | *CDH12* | rs6452086 | 22659899 | T | 0.1005 | 7.96E-07 |
| 5 | *CDH12* | rs6888927 | 22659781 | T | 0.1005 | 7.96E-07 |
| 5 | *CDH12* | rs3103162 | 22668926 | T | 0.1037 | 8.64E-07 |
| 5 | *CDH12* | rs268976 | 22668289 | G | 0.1034 | 9.26E-07 |
| 5 | *CDH12* | rs6452088 | 22663477 | A | 0.1001 | 9.41E-07 |
| 5 | *CDH12* | rs268974 | 22661481 | T | 0.1015 | 1.37E-06 |
| 5 | *CDH12* | rs11954951 | 22677995 | A | 0.09489 | 2.22E-06 |
| 5 | *CDH12* | rs10037678 | 22672142 | C | 0.09397 | 2.79E-06 |
| 5 | *CDH12* | rs6870865 | 22673195 | G | 0.09397 | 2.79E-06 |
| 5 | *CDH12* | rs11954983 | 22678125 | A | 0.09365 | 2.89E-06 |
| 5 | *CDH12* | rs1487409 | 22677440 | A | 0.09365 | 2.89E-06 |
| 5 | *CDH12* | rs10472484 | 22671430 | G | 0.09374 | 2.95E-06 |
| 3 | *STAC* | rs112766131 | 36422135 | A | -0.1107 | 2.96E-06 |
| 3 | *STAC* | rs76106538 | 36424274 | C | -0.1106 | 2.97E-06 |
| 3 | *STAC* | rs76699580 | 36433186 | A | -0.1102 | 3.25E-06 |
| 3 | *STAC* | rs1531134 | 36425536 | G | -0.1098 | 3.25E-06 |
| 3 | *STAC* | rs2361039 | 36429620 | T | -0.11 | 3.37E-06 |
| 10 | *RBM17* | rs1073646 | 6164310 | C | 0.09663 | 3.43E-06 |
| 5 | *CDH12* | rs2100939 | 22675249 | G | 0.09292 | 3.47E-06 |
| 5 | *CDH12* | rs9293029 | 22675489 | A | 0.09292 | 3.47E-06 |
| 5 | *CDH12* | rs10040534 | 22672259 | A | 0.09273 | 3.62E-06 |
| 5 | *CDH12* | rs10043072 | 22672276 | G | 0.09273 | 3.62E-06 |
| 5 | *CDH12* | rs13157365 | 22672810 | T | 0.09273 | 3.62E-06 |
| 5 | *CDH12* | rs1038591 | 22670144 | A | 0.09322 | 3.64E-06 |
| 3 | *STAC* | rs17034997 | 36432890 | G | -0.1096 | 3.73E-06 |
| 5 | *CDH12* | rs10473605 | 22671562 | G | 0.09251 | 3.82E-06 |
| 5 | *CDH12* | rs13185100 | 22671331 | A | 0.09251 | 3.82E-06 |
| 10 | *RBM17* | rs11256879 | 6163652 | A | 0.09691 | 3.94E-06 |
| 5 | *CDH12* | rs268960 | 22675740 | A | 0.09514 | 4.46E-06 |
| 5 | *CDH12* | rs174874 | 22712176 | T | 0.09535 | 5.05E-06 |
| 5 | *CDH12* | rs2169790 | 22670207 | T | 0.09162 | 5.19E-06 |
| 5 | *CDH12* | rs4299697 | 22670033 | T | 0.09162 | 5.19E-06 |
| 5 | *CDH12* | rs269030 | 22710970 | G | 0.09402 | 6.19E-06 |
| 5 | *CDH12* | rs269032 | 22711210 | C | 0.0936 | 6.88E-06 |
| 5 | *CDH12* | rs269033 | 22711440 | G | 0.0936 | 6.88E-06 |
| 5 | *CDH12* | rs269034 | 22712341 | T | 0.0936 | 6.88E-06 |
| 5 | *CDH12* | rs389000 | 22712137 | A | 0.0936 | 6.88E-06 |
| 5 | *CDH12* | rs12188885 | 22733475 | A | 0.0962 | 7.04E-06 |
| 5 | *CDH12* | rs72748717 | 22729360 | A | 0.09606 | 7.23E-06 |
| 5 | *CDH12* | rs12652487 | 22730470 | T | 0.09589 | 7.51E-06 |
| 5 | *CDH12* | rs7737131 | 22731563 | C | 0.09589 | 7.51E-06 |
| 5 | *CDH12* | rs269028 | 22710922 | T | 0.09308 | 7.93E-06 |
| 5 | *CDH12* | rs269029 | 22710928 | G | 0.09308 | 7.93E-06 |
| 5 | *CDH12* | rs12188256 | 22749678 | G | 0.09559 | 8.23E-06 |
| 5 | *CDH12* | rs72748721 | 22736128 | A | 0.09546 | 8.37E-06 |
| 5 | *CDH12* | rs269035 | 22712445 | T | 0.09265 | 8.65E-06 |
| 5 | *CDH12* | rs269036 | 22712675 | C | 0.09265 | 8.65E-06 |
| 5 | *CDH12* | rs3103161 | 22712896 | T | 0.09292 | 8.79E-06 |
| 5 | *CDH12* | rs269025 | 22710675 | T | 0.09287 | 8.86E-06 |
| 5 | *CDH12* | rs369274 | 22710662 | C | 0.09287 | 8.86E-06 |
| 5 | *CDH12* | rs429911 | 22710664 | G | 0.09287 | 8.86E-06 |
| 5 | *CDH12* | rs2355850 | 22666467 | C | 0.0887 | 8.90E-06 |
| 5 | *CDH12* | rs269037 | 22712857 | C | 0.09242 | 9.35E-06 |

***Supplementary Table 3.* SNPs with a suggestive significance (p≤1x10^-5^) for brooding as outcome.** Chr: chromosome; SNP: single-nucleotide polymorphism; bp: base position; A1: minor allele; p: p-value. Genes based on UCSC Genome Browser, GRCh37/hg19: <https://genome.ucsc.edu/>

**SNP-based results for reflection**

**
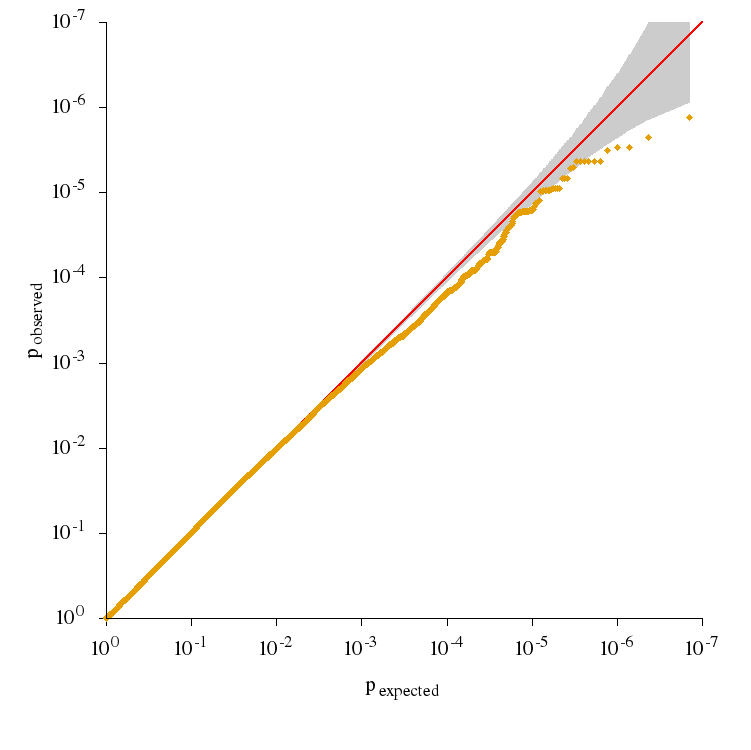
**

***Supplementary Figure 3*. Quantile-quantile plot of genome-wide SNP-based tests for reflection as outcome, with a 95% confidence interval marked.** SNP: single-nucleotide polymorphism.

| **Chr** | **Gene** | **SNP** | **Bp** | **A1** | **Beta** | **P** |
| --- | --- | --- | --- | --- | --- | --- |
| 12 | intergenic | rs137970858 | 73402019 | C | 0.4151 | 1.30E-06 |
| 12 | intergenic | rs76192797 | 74081159 | C | 0.2195 | 2.27E-06 |
| 12 | intergenic | rs117102221 | 73322538 | G | 0.3061 | 2.93E-06 |
| 12 | intergenic | rs148839335 | 73314999 | C | 0.312 | 2.93E-06 |
| 12 | intergenic | rs149573145 | 73410409 | T | 0.4038 | 3.19E-06 |
| 12 | intergenic | rs117331237 | 74096870 | C | 0.2122 | 4.28E-06 |
| 12 | intergenic | rs75365258 | 74113591 | T | 0.2122 | 4.28E-06 |
| 12 | intergenic | rs78252332 | 74114422 | C | 0.2122 | 4.28E-06 |
| 12 | intergenic | rs73338127 | 74095932 | T | 0.2122 | 4.33E-06 |
| 12 | intergenic | rs73338143 | 74103268 | C | 0.2122 | 4.33E-06 |
| 12 | intergenic | rs73338150 | 74106626 | T | 0.2122 | 4.33E-06 |
| 12 | intergenic | rs73338114 | 74080381 | T | 0.2143 | 5.11E-06 |
| 12 | intergenic | rs150616864 | 74065651 | T | 0.2584 | 5.14E-06 |
| 1 | *CHRM3* | rs10925907 | 239698862 | G | -0.0948 | 6.82E-06 |
| 2 | *DPYSL5* | rs12470698 | 27063261 | G | -0.0882 | 6.88E-06 |
| 2 | *DPYSL5* | rs12474330 | 27063260 | A | -0.0882 | 6.88E-06 |
| 12 | intergenic | rs117168252 | 74058074 | A | 0.2424 | 8.80E-06 |
| 12 | intergenic | rs79547406 | 74059813 | C | 0.2424 | 8.80E-06 |
| 12 | intergenic | rs139629289 | 74000969 | C | 0.2422 | 8.89E-06 |
| 12 | intergenic | rs138039151 | 74068300 | T | 0.2424 | 8.97E-06 |
| 12 | intergenic | rs10450801 | 74056572 | T | 0.2419 | 9.15E-06 |
| 12 | intergenic | rs116990743 | 74003269 | A | 0.2417 | 9.33E-06 |
| 12 | intergenic | rs117622404 | 74001671 | G | 0.2417 | 9.33E-06 |
| 12 | intergenic | rs11834804 | 74006047 | T | 0.2417 | 9.33E-06 |
| 12 | intergenic | rs17112553 | 74011985 | T | 0.2417 | 9.33E-06 |
| 12 | intergenic | rs2363689 | 74018237 | C | 0.2415 | 9.45E-06 |
| 12 | intergenic | rs116017074 | 74042780 | T | 0.2414 | 9.67E-06 |
| 12 | intergenic | rs76507690 | 74043451 | A | 0.2414 | 9.67E-06 |

***Supplementary Table 4.* SNPs with a suggestive significance (p≤1x10^-5^) for reflection as outcome**. Chr: chromosome; SNP: single-nucleotide polymorphism; bp: base position; A1: minor allele; p: p-value. Genes based on UCSC Genome Browser, GRCh37/hg19: <https://genome.ucsc.edu/>

**
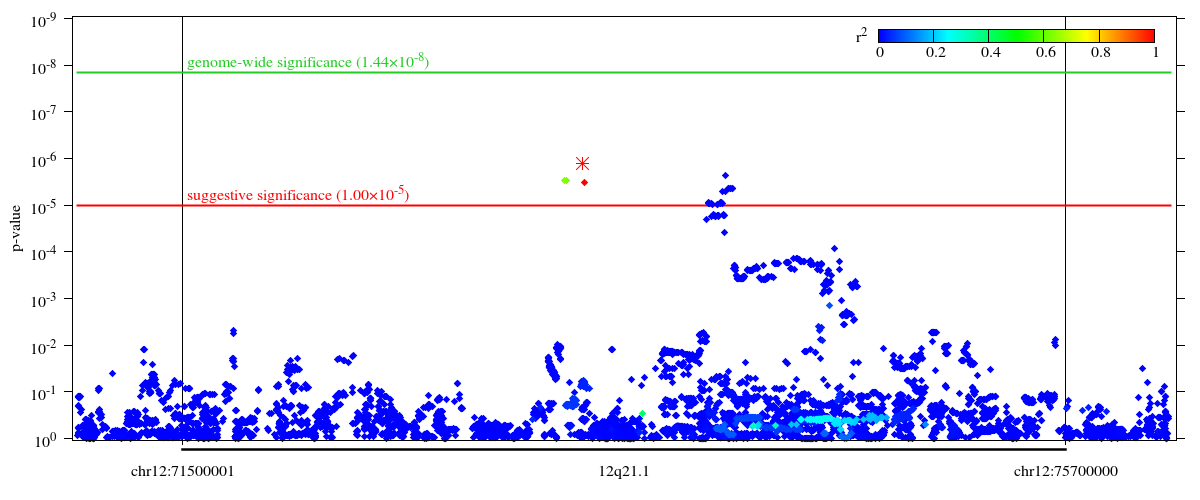
**

***Supplementary Figure 4.* Zoomed Manhattan plot of the 12q21.1 region for reflection as outcome.** P-value is displayed in function of genomic position for each single-nucleotide polymorphism (SNP) in the region. Colors denote the r^2^ value of linkage disequilibrium (LD) with the most significant SNP (marked with asterisk).

**Genes regulated in brain tissues by top SNPs of rumination**


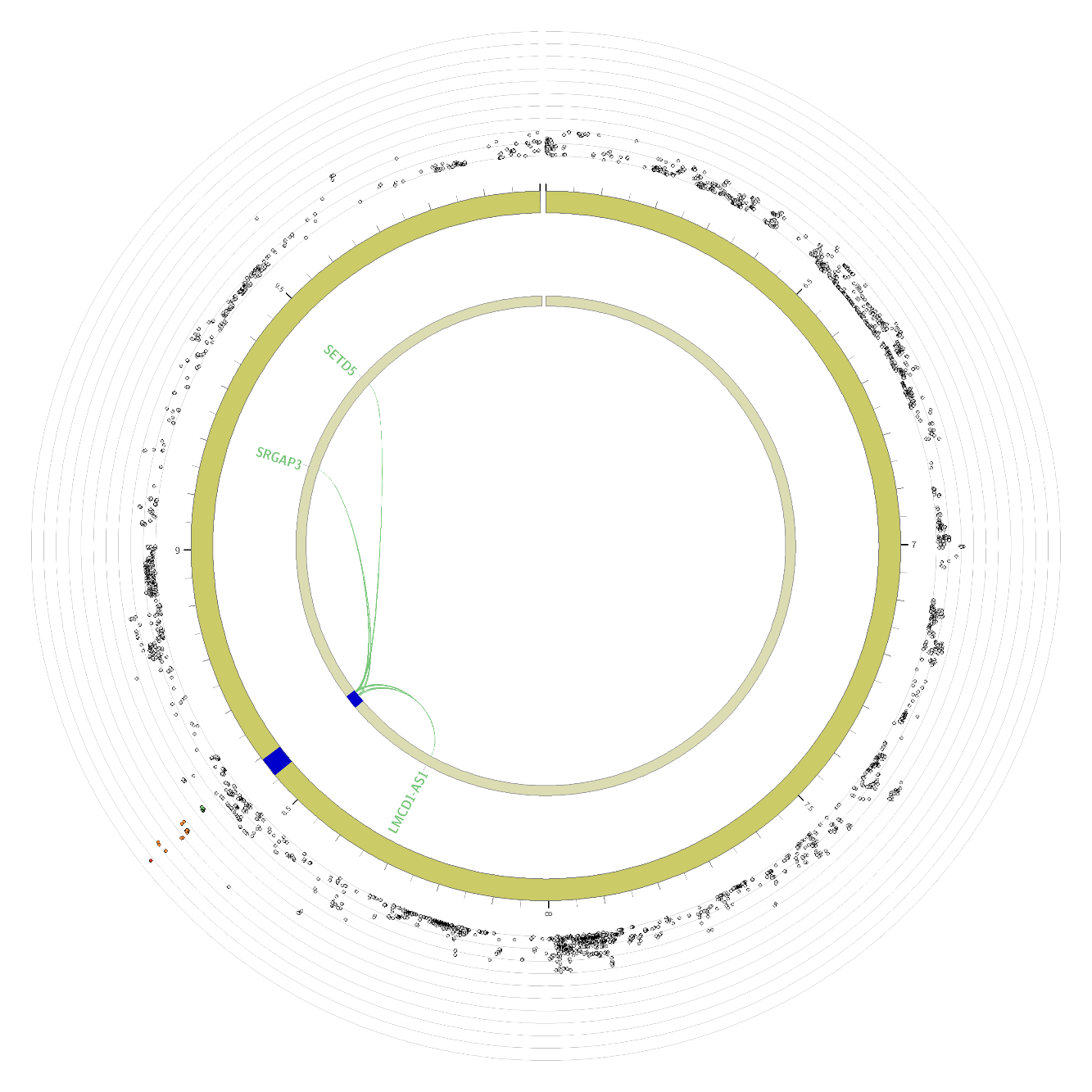


***Supplementary Figure 5.* Circos plot of brain gene regulatory role of our top SNPs for rumination on chromosome 3.** Inside the zoomed Manhattan plot of SNPs with p<0.05 for rumination and genomic risk loci marked with blue, green color denotes links and mapped genes based on eQTL, and orange color denotes links and mapped genes based on chromatin interaction external databases. Red color denotes genes mapped by both regulatory mechanisms. SNP: single-nucleotide polymorphism; eQTL: expression quantitative trait loci.


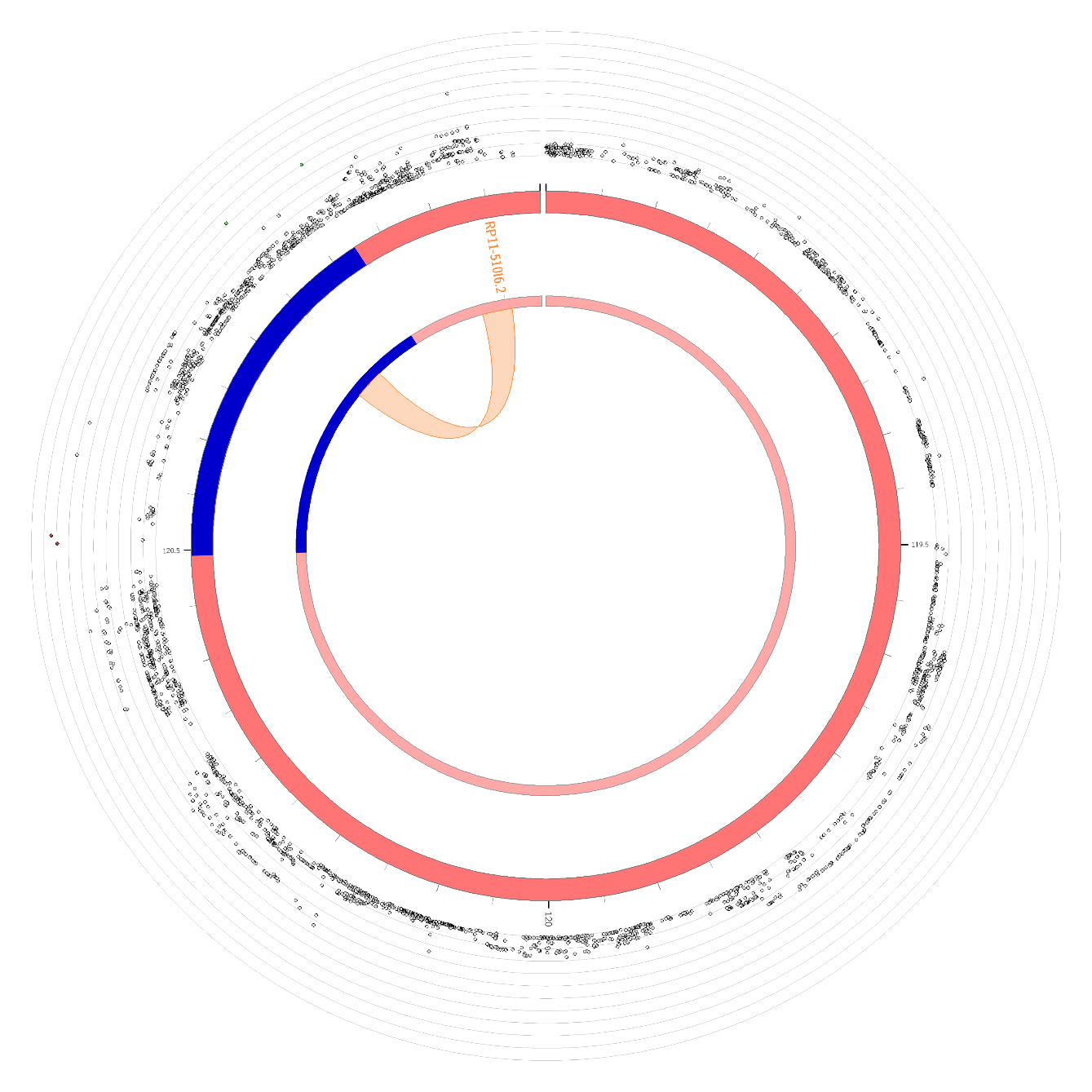


***Supplementary Figure 6.* Circos plot of brain gene regulatory role of our top SNPs for rumination on chromosome 5.** Inside the zoomed Manhattan plot of SNPs with p<0.05 for rumination and genomic risk loci marked with blue, green color denotes links and mapped genes based on eQTL, and orange color denotes links and mapped genes based on chromatin interaction external databases. Red color denotes genes mapped by both regulatory mechanisms. SNP: single-nucleotide polymorphism; eQTL: expression quantitative trait loci.


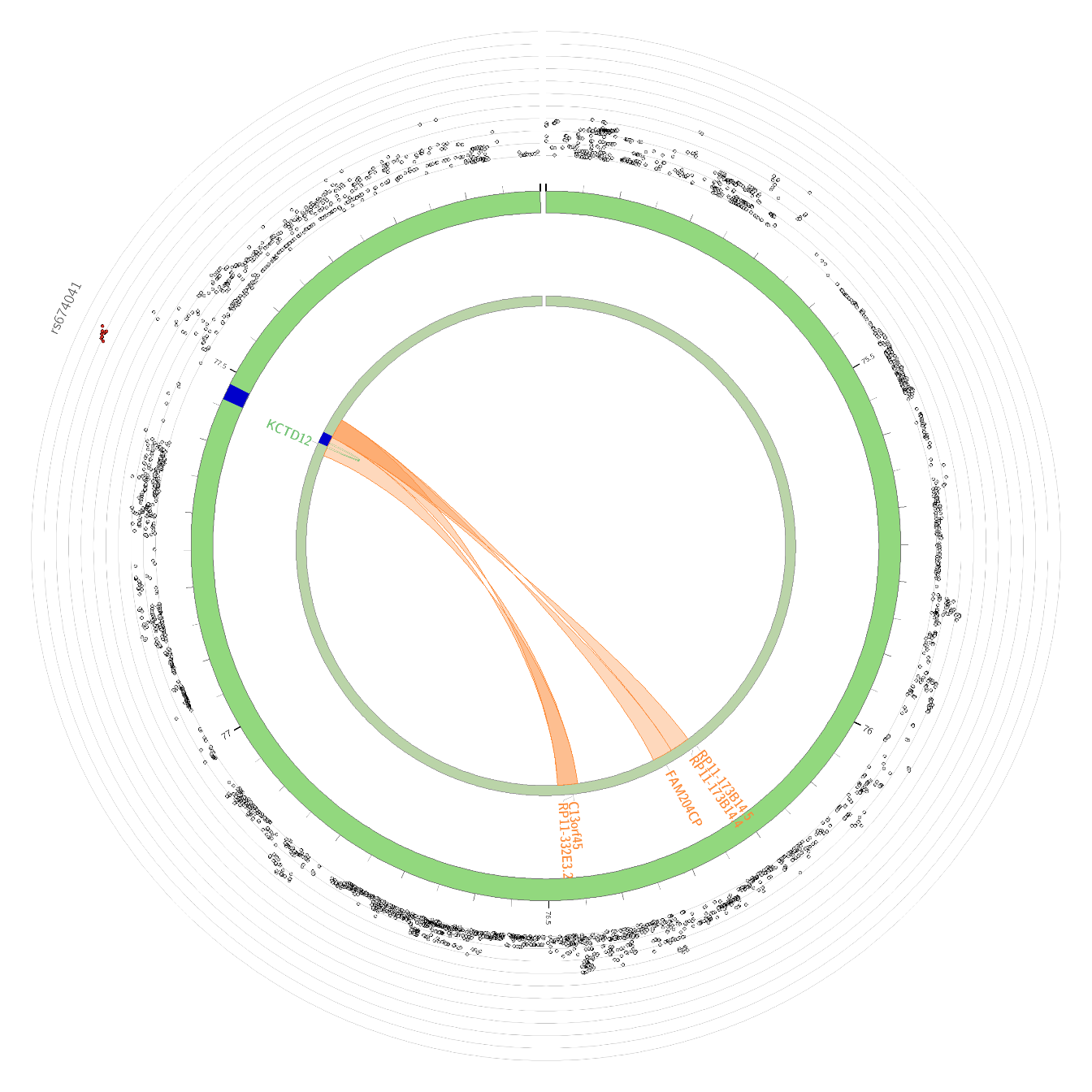


***Supplementary Figure 7.* Circos plot of brain gene regulatory role of our top SNPs for rumination on chromosome 13.** Inside the zoomed Manhattan plot of SNPs with p<0.05 for rumination and genomic risk loci marked with blue, green color denotes links and mapped genes based on eQTL, and orange color denotes links and mapped genes based on chromatin interaction external databases. Red color denotes genes mapped by both regulatory mechanisms. SNP: single-nucleotide polymorphism; eQTL: expression quantitative trait loci.

**Genes regulated in brain tissues by top SNPs of brooding**


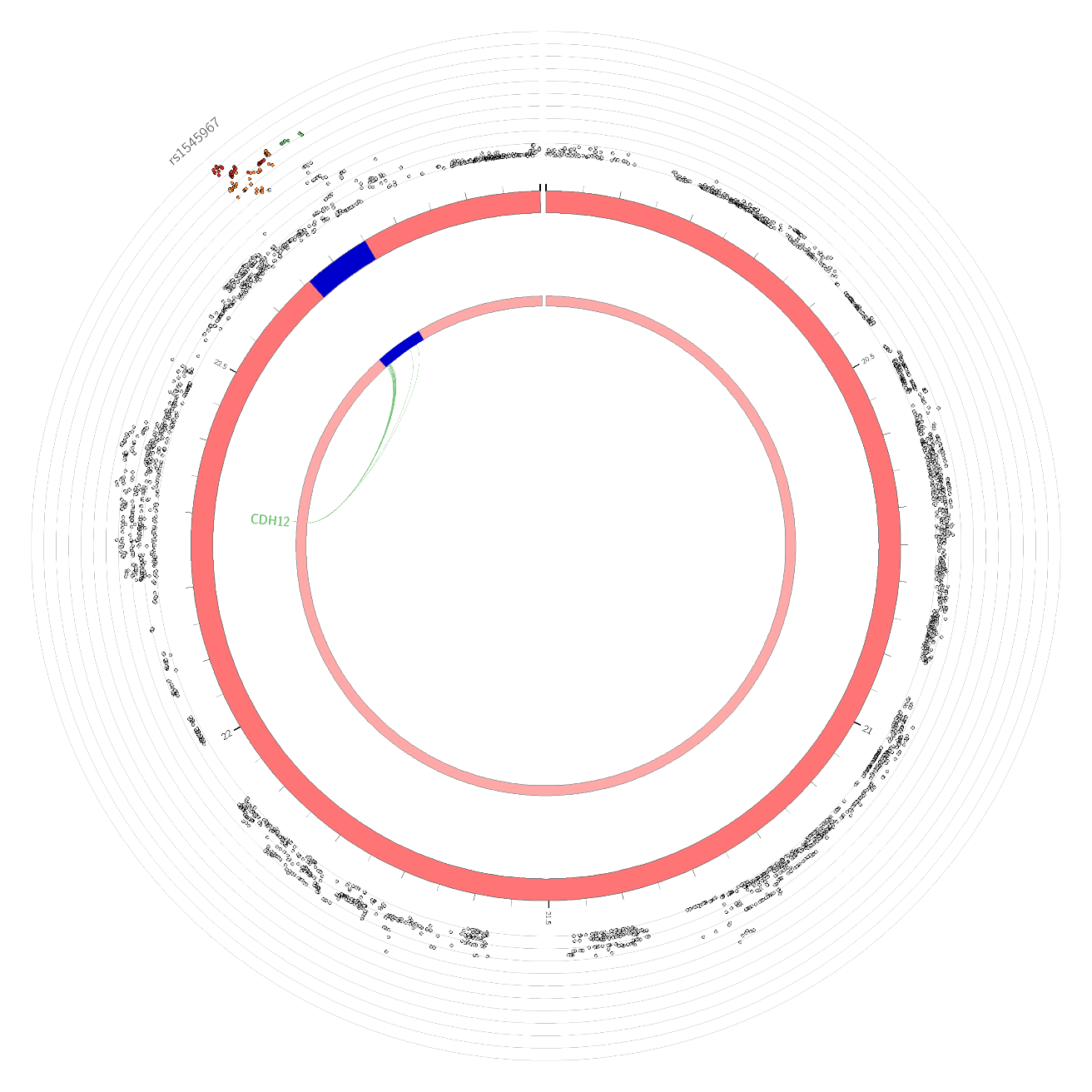


***Supplementary Figure 8.* Circos plot of brain gene regulatory role of our top SNPs for brooding on chromosome 5.** Inside the zoomed Manhattan plot of SNPs with p<0.05 for brooding and genomic risk loci marked with blue, green color denotes links and mapped genes based on eQTL, and orange color denotes links and mapped genes based on chromatin interaction external databases. Red color denotes genes mapped by both regulatory mechanisms. SNP: single-nucleotide polymorphism; eQTL: expression quantitative trait loci.

**Genes regulated in brain tissues by top SNPs of reflection**

**
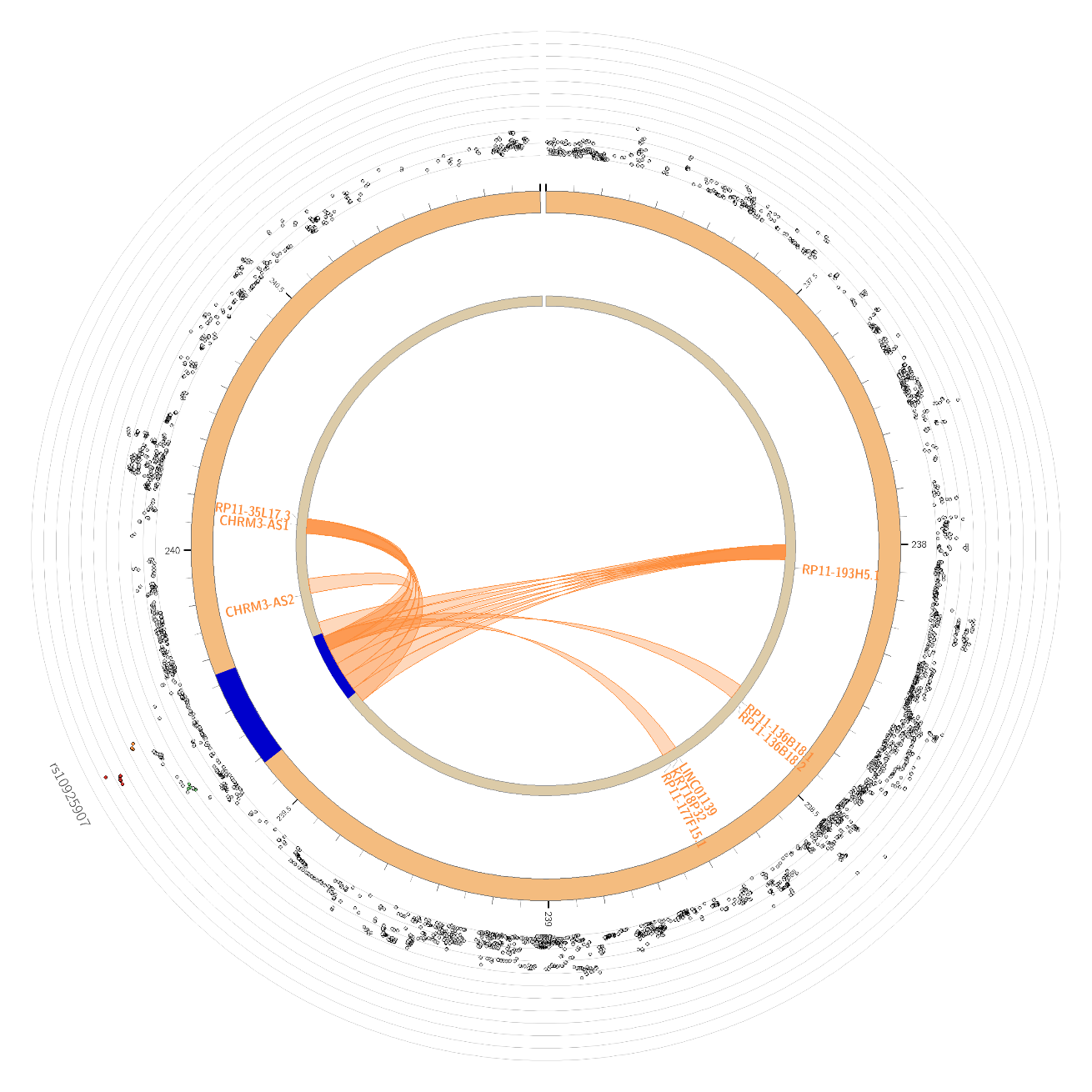
**

***Supplementary Figure 9.* Circos plot of brain gene regulatory role of our top SNPs for reflection on chromosome 1.** Inside the zoomed Manhattan plot of SNPs with p<0.05 for reflection and genomic risk loci marked with blue, green color denotes links and mapped genes based on eQTL, and orange color denotes links and mapped genes based on chromatin interaction external databases. Red color denotes genes mapped by both regulatory mechanisms. SNP: single-nucleotide polymorphism; eQTL: expression quantitative trait loci.

**
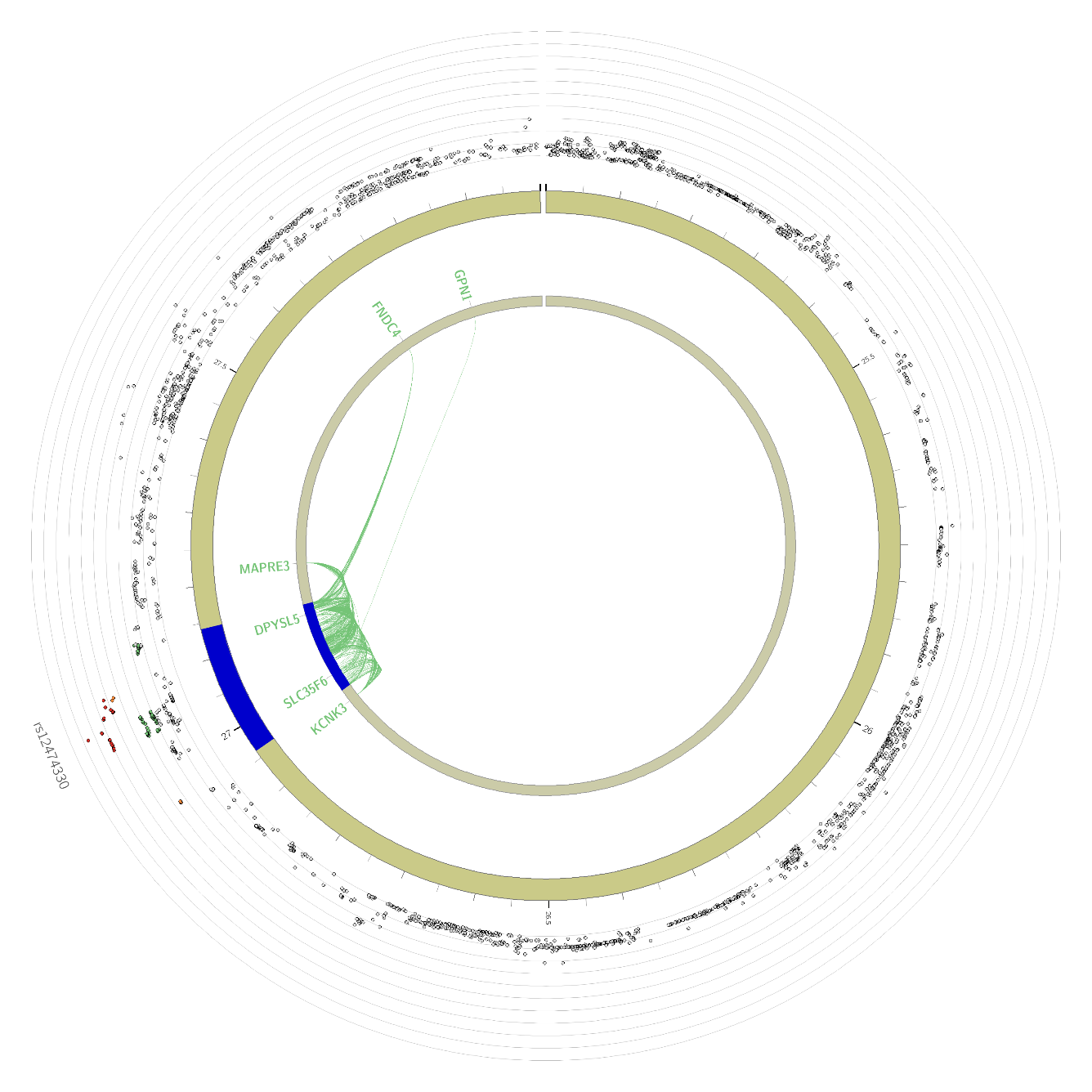
**

***Supplementary Figure 10.* Circos plot of brain gene regulatory role of our top SNPs for reflection on chromosome 2.** Inside the zoomed Manhattan plot of SNPs with p<0.05 for reflection and genomic risk loci marked with blue, green color denotes links and mapped genes based on eQTL, and orange color denotes links and mapped genes based on chromatin interaction external databases. Red color denotes genes mapped by both regulatory mechanisms. SNP: single-nucleotide polymorphism; eQTL: expression quantitative trait loci.


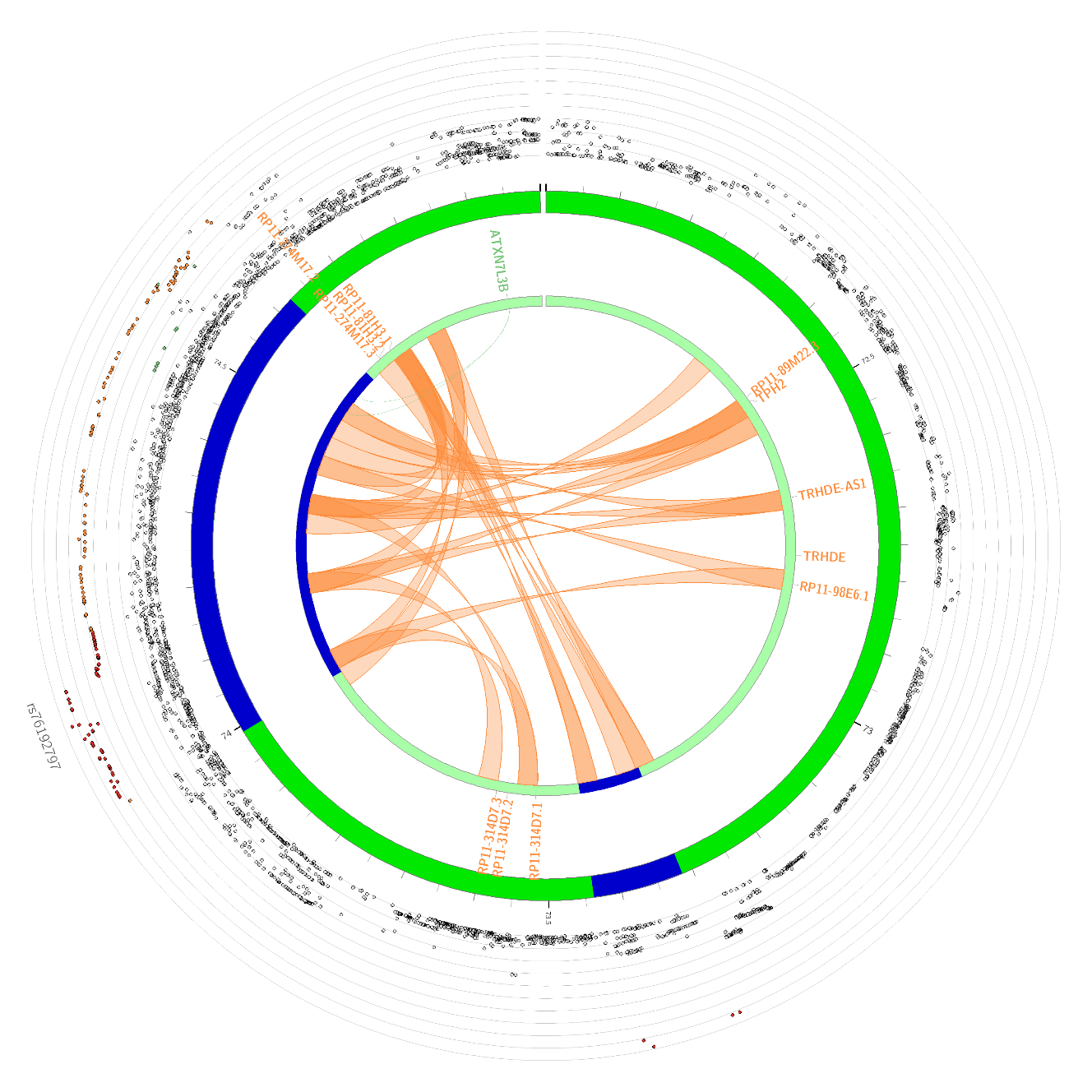


***Supplementary Figure 11.* Circos plot of brain gene regulatory role of our top SNPs for reflection on chromosome 12.** Inside the zoomed Manhattan plot of SNPs with p<0.05 for reflection and genomic risk loci marked with blue, green color denotes links and mapped genes based on eQTL, and orange color denotes links and mapped genes based on chromatin interaction external databases. Red color denotes genes mapped by both regulatory mechanisms. SNP: single-nucleotide polymorphism; eQTL: expression quantitative trait loci.
