# Supplementary material for: Genome-wide association analysis reveals KCTD12 and miR-383-binding genes in the background of rumination
Source: Transl Psychiatry. 2019 Mar 18;9:119. doi: 10.1038/s41398-019-0454-1 (PMC6423133; doi:10.1038/s41398-019-0454-1)
Supplement: Supplementary file 5 — Supplemental legends [file 41398_2019_454_MOESM5_ESM.docx]

Supplemental Information

Supplementary File 1: supplementary text, figures and tables

Supplementary File 2: detailed results for rumination

Supplementary File 3: detailed results for brooding

Supplementary File 4: detailed results for reflection
